# The Role of the Multi-Inflammatory Index as a Novel Predictor of Hospital Mortality in Acute Ischemic Stroke

**DOI:** 10.7759/cureus.43258

**Published:** 2023-08-10

**Authors:** Mustafa E Demirel, Canan Akunal Türel

**Affiliations:** 1 Emergency Medicine, Abant Izzet Baysal University Hospital, Bolu, TUR; 2 Neurology, Abant Izzet Baysal University Hospital, Bolu, TUR

**Keywords:** patient outcomes, assessment, ischemic stroke, mortality, multi-inflammatory index

## Abstract

Background and objective

Ischemic strokes account for the majority of all strokes. The severity of an acute ischemic stroke (AIS) can be estimated with the help of a number of different scoring systems. However, there is a need for bedside tests that will support the clinical diagnosis and thus help predict the severity of stroke. The research on the multi-inflammatory index (MII), which is calculated using hemogram parameters, has shown immense promise. In light of this, the aim of this study was to establish the association between MII and the severity of AIS.

Methods

The study included 452 ischemic stroke patients over the age of 18 years who presented to the hospital within 72 hours of the onset of symptoms. Demographic information such as patient age and gender, hemogram parameters, ratios, indices, hospitalization, and mortality status were all recorded. The demographic data, hemogram parameters, neutrophil/lymphocyte ratio (NLR), platelet/lymphocyte ratio (PLR), and C‐reactive protein (CRP)/lymphocyte ratio (CLR), and MII 1, 2, and 3 were compared between surviving and deceased patients.

Results

The MII-1, MII-2, and MII-3 index values were determined to be significantly low in the patients with Glasgow Coma Scale (GCS) scores of 13-15 compared to those with GCS scores ≤8, and in patients with National Institutes of Health Stroke Scale (NIHSS) score of 1-4 compared to those with scores of 5-14, 15-20, and ≥21. The NLR, CLR, PLR, MII-1, MII-2, and MII-3 index values were significantly higher in the non-survivors (PLR: p=0.004, all other values: p<0.001). The performances of multiple models developed for the mortality cut-off points were evaluated. Together with other factors, Model 1 included the MII-1, Model 2 the MII-2, and Model 3 the MII-3. Although there was no significant difference between the AUC values of the models, the highest sensitivity rate was reached with Model 2 (74.48%), and the highest specificity rate with Model 3 (90.62%).

Conclusion

Based on our findings, MII is a simple and practical biomarker that can be easily obtained from NLR, PLR, and CRP, and can help in the early detection of poor prognosis in AIS. NLR was found to be superior to PLR and CLR in distinguishing fatal AIS cases.

## Introduction

Stroke is a type of acute neurological dysfunction caused by hemorrhage or ischemia in the central nervous system and is one of the leading causes of mortality and disability worldwide. Ischemic strokes account for approximately 80% of all strokes [1]. The severity of acute ischemic stroke (AIS) can be estimated using a number of different scoring systems, such as the Glasgow Coma Scale (GCS) and the National Institutes of Health Stroke Scale (NIHSS). The GCS and NIHSS are widely used neurological scales that provide a reliable and objective way to record the stroke patient's state of consciousness during initial and subsequent assessments and can provide vital information about the likelihood of recovery [2,3].

Inflammatory events, as well as other risk factors for ischemic stroke, play an important role in the pathophysiology of cerebral ischemia. Over the past 20 years, several inflammation biomarkers have been found to be associated with cardiovascular, cerebrovascular, psychiatric, and other events [4-6]. Determining risk factors that will help predict poor outcomes after stroke is critical since it enables the development of preventive strategies and implementation of fast management when needed. With advancements in stroke care, there is a need for a bedside test that predicts stroke severity and supports the clinical diagnosis, thereby assisting prognosis. Many risk factors for poor outcomes have been identified, and various risk indexing and modeling studies have also been conducted in the literature. The result of increased inflammation during the clinical course suggests that indices including the neutrophil-lymphocyte ratio (NLR), platelet-lymphocyte ratio (PLR), and CRP levels may help in determining prognosis and disease severity. In addition, research has begun on the multi-inflammatory index (MII), which is calculated using hemogram parameters. A novel relationship has recently been reported between MII and the prognosis and clinical severity of inflammatory-related cancers, pulmonary events, and viral diseases [7-9]. MII's predictive value for stroke severity and mortality has not yet been evaluated. In this study, we aimed to investigate the role of multi-inflammatory indices, which can be calculated by hemogram parameters, in the prediction of mortality in the early period.

## Materials and methods

Study population

Patients over the age of 18 years who were diagnosed with AIS and admitted to the hospital within 72 hours of the onset of symptoms were included in the study. All patients were newly diagnosed with AIS based on clinical symptoms and neuroimages listed in the World Health Organization (WHO) criteria [10]. Patients with a diagnosis other than AIS such as trauma, tumor, infection, immunosuppressives, and hepatic and renal dysfunction were excluded [11,12]. We adopted a retrospective study design. Approval for the study was granted by the Bolu Abant Izzet Baysal University Clinical Researches Ethics Committee (2022/273; date: 25.10.2022).

Demographic and clinical information such as age and gender, hemogram parameters, the Modified Rankin Score (mRS), Trial of ORG 10172 in Acute Stroke Treatment (TOAST), and Bamford classifications, ratios, indices, and hospitalization and the mortality status of the patients were recorded. Demographic characteristics, hemogram parameters, ratios, and inflammatory indices were compared between surviving and non-surviving patients. Indexes and scoring were calculated based on patients' biochemical parameters and examination findings at the time of admission.

MII-1, MII-2, and MII-3 were defined using the following formulas [8]: MII-1: NLR x CRP; MII-2: PLR x CRP; MII-3: (platelet x neutrophil/lymphocyte) x CRP.

Study data

The sociodemographic and clinical characteristics of patients admitted to the hospital between January 2021 and March 2022 due to AIS were collected on a data form. The demographic characteristics of the patients (age, gender), clinical history (history, family history, and medications), clinical characteristics, results of laboratory tests performed during admission to the emergency department, and brain imaging results (brain CT and diffusion MRI) were documented in the data collection form. Neurological assessments were made using the NIHSS, and stroke-related disability was assessed using the mRS [13]. According to the NIHSS, scores of 1-4 were considered mild, 5-14 moderate, and 15-20 moderate-severe, while scores ≥21 indicated severe paralysis. An mRS score of 0-2 was indicative of mild stroke and 3-6 of severe stroke.

Statistical analysis

The normality condition for continuous variables was checked with the Shapiro-Wilk test. Continuous data were compared with the Mann-Whitney U test between the two groups. Relationships between three or more groups were examined with the Kruskal-Wallis test. Relationships between two categorical variables were examined with Pearson's Chi-squared test or Fisher's exact test. Receiver operating characteristics (ROC) (in RStudio - pROC package v. 1.17.0.1) analysis was performed. AUC values were evaluated as follows: 0.9-1: excellent, 0.8-0.9: good, 0.7-0.8: fair, 0.6-0.7: poor, and <0.6: not acceptable/failed [14-16]. Continuous data were divided into two groups according to the cut-off values determined using the Youden Index. Sensitivity and specificity performance measures were calculated. Simple logistic regression analysis was applied for mortality. Parameters with a result of p<0.20 from the simple analysis were included in the multiple analysis (Forward Wald method). Before the multiple analysis, models were created considering the correlation level between parameters (r<0.70) to avoid any multicollinearity problem. The Akaike information criterion (AIC) was used to select the best model from the models created for each index (MII-1, 2, and 3). AUC values and cut-off points were determined for the estimated probabilities of the created models, and sensitivity and specificity measures were calculated. The AUC values of the models were compared with the DeLong test. Statistical analyses were performed using IBM SPSS Statistics software version 23 (IBM Corp., Armonk, NY). The level of statistical significance was set at p<0.05.

## Results

In this study, 452 ischemic stroke patients were examined. The median age of the patients was 73 years (64-83) and 51.11% (n: 231) were male. The most common comorbidities were hypertension (n: 247, 54.65%), diabetes mellitus (DM) (n: 136, 30.09%), atrial fibrillation (AF) (n: 125, 27.65%), coronary artery disease (n: 110, 24.34%), and myocardial infarction/cerebrovascular event (MI/CVE) (n: 106, 23.45%), respectively. GCS score ≤8 was observed in 16.59% (n: 75) of the patients and GCS of 9-12 in 31.64% (n: 143). The NIHSS scores of 5-14, 15-20, and ≥21 were found in 47.79% (n: 216), 19.03% (n: 86), and 6.86% (n: 31) of the patients, respectively. The mRS, TOAST, and Bamford classifications of the patients are presented in Table 1.

**Table 1:** General characteristics IQR: interquartile range; MI/CVE: myocardial infarction/cerebrovascular event; AF: atrial fibrillation; PAD: peripheral artery disease; GCS: Glasgow Coma Scale; NIHSS: National Institutes of Health Stroke Scale; mRS: Modified Rankin Score; TOAST: Trial of ORG 10172 in Acute Stroke Treatment; TACI: total anterior circulation infarcts; PACI: partial anterior circulation infarcts; POCI: posterior circulation infarcts; LACI: lacunar infarcts

Variables	Values (n=452)
Age, years, median (IQR)	73 (64-83)
Gender - male, n (%)	231 (51.11)
Smoker, n (%)	66 (14.6)
Comorbidities, n (%)	
Hypertension	247 (54.65)
Diabetes	136 (30.09)
Hyperlipidemia	39 (8.63)
Coronary artery disease	110 (24.34)
MI/CVE	106 (23.45)
AF	125 (27.65)
PAD	1 (0.22)
Chronic renal failure	11 (2.43)
GCS score, n (%)	
≤8	75 (16.59)
9-12	143 (31.64)
13-15	234 (51.77)
NIHSS score, n (%)	
1-4	119 (26.33)
5-14	216 (47.79)
15-20	86 (19.03)
≥21	31 (6.86)
mRS, n (%)	
Mild (0-2)	146 (32.3)
Moderate (3-4)	172 (38.05)
Severe (5-6)	134 (29.65)
TOAST, n (%)	
Large artery atherosclerosis	205 (45.35)
Cardioembolism	121 (26.77)
Small vascular obstruction	33 (7.3)
Other etiology	44 (9.73)
No known cause	49 (10.84)
Bamford, n (%)	
TACI	94 (20.8)
PACI	240 (53.1)
POCI	99 (21.9)
LACI	19 (4.2)

**Figure 1:**
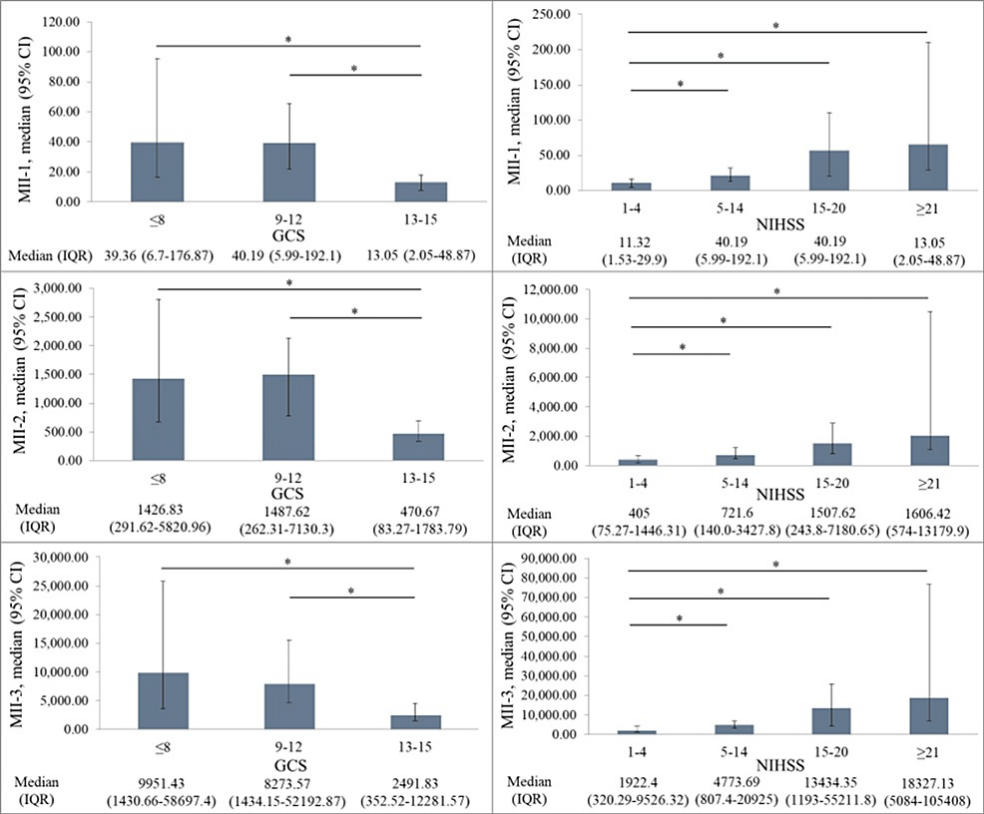
Comparisons of the MII-1, MII-2, and MII-3 indexes according to GCS and NIHSS scores *Kruskal-Wallis H test: p<0.00, Adj. Dunn test: p<0.05 GCS: Glasgow Coma Scale; NIHSS: National Institutes of Health Stroke Scale

The MII-1, MII-2, and MII-3 index values were determined to be significantly low in the patients with GCS scores of 13-15 compared to those with GCS ≤8, and in the patients with NIHSS scores of 1-4 compared to those with scores of 5-14, 15-20, and ≥21 (p<0.001 for both, Figure 1).

The general characteristics, laboratory values, and index values of the patients were examined in terms of mortality. The results of these examinations are shown in Table 2.

**Table 2:** Comparison of patient characteristics with respect to mortality ^a,b,c^Significant differences between groups according to the post-Hoc test (Adj. Bonferroni test) results are indicated with different letters, p<0.05 Continuous variables are presented as median (IQR, 25th-75th percentile), and categorical variables as n (%) and were compared with the Mann-Whitney U test, Pearson's Chi-squared test, and Fisher’s exact test, respectively MI/CVE: myocardial infarction/cerebrovascular event; AF: atrial fibrillation; PAD: peripheral artery disease; GCS: Glasgow Coma Scale; NIHSS: National Institutes of Health Stroke Scale; TOAST: Trial of ORG 10172 in Acute Stroke Treatment; TACI: total anterior circulation infarcts; PACI: partial anterior circulation infarcts; POCI: posterior circulation infarcts; LACI: lacunar infarcts; WBC: white blood cells; HGB: hemoglobin; PLT: platelets; LYM: lymphocytes; MONO: monocytes; NEU: neutrophils; EOS: eosinophils; CRP: C-reactive protein; NLR: neutrophil/lymphocyte ratio; CLR: C‐reactive protein/lymphocyte ratio; PLR: platelet/lymphocyte ratio; MII-1: multi-inflammatory index 1; MII-2: multi-inflammatory index 2; MII-3; multi-inflammatory index 3

Characteristics	Mortality		P-value
	Survivors (n=373)	Non-survivors (n=79)	
Age, years	73 (64-83)	76 (63-83)	0.629
Gender			
Female	171 (77.38)	50 (22.62)	0.005
Male	202 (87.45)	29 (12.55)	
Smoking			
Non-smoker	319 (82.64)	67 (17.36)	0.871
Smoker	54 (81.82)	12 (18.18)	
Comorbidities			
Hypertension	201 (81.38)	46 (18.62)	0.481
Absent	172 (83.9)	33 (16.1)	
Diabetes	99 (72.79)	37 (27.21)	<0.001
Absent	274 (86.71)	42 (13.29)	
Hyperlipidemia	35 (89.74)	4 (10.26)	0.214
Absent	338 (81.84)	75 (18.16)	
Coronary artery disease	93 (84.55)	17 (15.45)	0.521
Absent	280 (81.87)	62 (18.13)	
MI/CVE	93 (87.74)	13 (12.26)	0.106
Absent	280 (80.92)	66 (19.08)	
AF	103 (82.4)	22 (17.6)	0.966
Absent	270 (82.57)	57 (17.43)	
PAD	0 (0)	1 (100)	-
Absent	373 (82.71)	78 (17.29)	
Chronic renal failure	5 (45.45)	6 (54.55)	0.005*
Absent	368 (83.45)	73 (16.55)	
GCS score			
≤8	27 (36)^a^	48 (64)^a^	<0.001
9-12	123 (86.01)^b^	20 (13.99)^b^	
13-15	223 (95.3)^c^	11 (4.7)^c^	
NIHSS score			
1-4	117 (98.32)^a^	2 (1.68)^a^	<0.001
5-14	199 (92.13)^a^	17 (7.87)^a^	
15-20	47 (54.65)^b^	39 (45.35)^b^	
≥21	10 (32.26)^b^	21 (67.74)^b^	
TOAST			
Large artery atherosclerosis	164 (80)	41 (20)	0.261
Cardioembolism	98 (80.99)	23 (19.01)	
Small vascular obstruction	31 (93.94)	2 (6.06)	
Other etiology	39 (88.64)	5 (11.36)	
No known cause	41 (83.67)	8 (16.33)	
Bamford			
TACI	51 (54.26)	43 (45.74)	<0.001
PACI	214 (89.17)	26 (10.83)	
POCI	89 (89.9)	10 (10.1)	
LACI	19 (100)	0 (0)	
Laboratory values			
WBC, 10^3^/μL	8.65 (6.79-10.97)	11.44 (8.49-14.68)	<0.001
HGB, g/dL	13.3 (12.2-14.9)	12.4 (11.3-14.5)	0.002
PLT, 10^3^/μL	231 (188-281)	251 (204-362)	<0.001
LYM, 10^3^/μL	1.65 (1.12-2.2)	1.56 (1-2.21)	0.501
MONO, 10^3^/μL	0.6 (0.47-0.78)	0.75 (0.52-0.94)	<0.001
NEU, 10^3^/μL	5.78 (4.12-8.31)	8.67 (5.45-11.69)	<0.001
EOS, 10^3^/μL	0.11 (0.04-0.2)	0.06 (0.01-0.12)	0.001
CRP, mg/L	5.4 (1-15.2)	12.5 (4.2-46.1)	<0.001
Indexes			
NLR	3.49 (2.17-5.87)	5.27 (3.54-9.92)	<0.001
CLR	2.73 (0.54-10.51)	7.59 (1.73-37.72)	<0.001
PLR	138.75 (100.88-199.57)	161.67 (114.41-294.42)	0.004
MII-1	16.74 (2.72-71.07)	78.19 (11.61-244.24)	<0.001
MII-2	586.79 (113.68-2400.09)	1821.9 (401.9-13811.15)	<0.001
MII-3	3615.14 (563.6-17029.8)	18944.4 (2577.7-88415.8)	<0.001
Length of stay in hospital, days	5 (3-9)	6 (3-14)	0.086

The mortality rates were higher in patients with the comorbid diseases of DM (DM vs. non-DM) and chronic renal failure (CRF) (CRF vs. non-CRF) (p<0.001, p=0.005). Mortality occurred in 17.48% (n: 79) of the patients, and it was at a higher rate in females (n: 50, 22.62%) than in males (n: 29, 12.55%) (p=0.005). A significant difference was found between all the GCS groups (GCS <8, 9-12, and 13-15) with respect to mortality, and the difference was significant for NIHSS scores between 15-20 and ≥21 groups and between the 1-4 and 5-14 groups (p<0.001 for both). The mortality rate was higher in the total anterior circulation infarcts (TACI) group compared to the other groups [partial anterior circulation infarcts (PACI): n: 26, 10.83%; posterior circulation infarcts (POCI): n: 10, 10.1%; lacunar infarcts (LACI): n: 0, 0% (p<0.001)]. When the laboratory values were examined, the white blood cells (WBC), platelets (PLT), monocytes (MONO), neutrophils (NEU), and CRP values were determined to be significantly higher in the patients who developed mortality (p<0.001 for all). The hemoglobin (HGB) and eosinophils (EOS) values were lower in the non-survivors (p=0.002, p=0.001, respectively). The NLR, CLR, PLR, MII-1, MII-2, and MII-3 values were significantly higher in the non-survivors (PLR: p=0.004, all other values: p<0.001).

The strength of the parameters in the differentiation of mortality was examined with ROC analysis, and all the parameters were seen to be “poor” (AUC: 0.6-0.7). The cut-off points determined with AUC and the sensitivity and specificity values calculated are shown in Table 3.

**Table 3:** ROC analysis results and performance measures for mortality ROC: receiver operating characteristic; AUC: area under the curve; CI: confidence interval; WBC: white blood cells; HGB: hemoglobin; PLT: platelets; LYM: lymphocytes; MONO: monocytes; NEU: neutrophils; EOS: eosinophils; CRP: C-reactive protein; NLR: neutrophil/lymphocyte ratio; CLR: C‐reactive protein/lymphocyte ratio; PLR: platelet/lymphocyte ratio; MII-1: multi-inflammatory index 1; MII-2: multi-inflammatory index 2; MII-3; multi-inflammatory index 3

Variables	AUC (95% CI)	Cut-off point	Sensitivity, % (95% CI)	Specificity, % (95% CI)
WBC, 10^3^/μL	0.69 (0.62-0.76)	11.245	54.43 (35.44-81.01)	79.36 (50.13-93.03)
HGB, g/dL	0.61 (0.54-0.68)	12.75	60.76 (44.3-75.95)	67.56 (53.62-78.02)
PLT, 10^3^/μL	0.62 (0.54-0.69)	326	36.71 (22.78-78.48)	90.35 (46.37-95.98)
MONO, 10^3^/μL	0.62 (0.54-0.69)	0.805	50.63 (29.11-75.95)	75.6 (49.06-88.2)
EOS, 10^3^/μL	0.62 (0.55-0.69)	0.45	56.96 (32.88-83.54)	68.9 (40.75-87.4)
NEU, 10^3^/μL	0.68 (0.61-0.75)	9.71	55.7 (37.97-78.48)	79.09 (53.62-89.54)
CRP, mg/dL	0.65 (0.58-0.72)	6.85	65.82 (32.88-81.01)	62.47 (46.38-90.62)
NLR	0.64 (0.57-0.71)	3.535	69.62 (40.51-84.81)	56.3 (47.45-84.99)
CLR	0.64 (0.57-0.71)	3.235	64.56 (34.18-84.81)	61.66 (39.14-87.41)
PLR	0.60 (0.53-0.68)	245.24	39.24 (25.32-82.28)	84.45 (39.14-92.49)
MII-1	0.67 (0.60-0.74)	32.095	64.56 (45.57-77.22)	67.02 (57.1-81.5)
MII-2	0.66 (0.59-0.73)	888.95	68.35 (27.85-81.01)	59.52 (51.21-95.98)
MII-3	0.68 (0.62-0.75)	17528.4	62.03 (46.84-78.48)	73.73 (57.37-81.5)

The parameters were separated into two groups according to the cut-off point determined with ROC analysis. These parameters were examined as two types of continuous and categorical variables in the logistic regression analyses. The simple logistic regression and multiple linear regression results related to mortality are shown in Table 4.

**Table 4:** Simple logistic regression and multiple logistic regression results for mortality OR: odds ratio; CI: confidence interval; MI/CVE: myocardial infarction/cerebrovascular event; AF: atrial fibrillation; GCS: Glasgow Coma Scale; NIHSS: National Institutes of Health Stroke Scale; TOAST: Trial of ORG 10172 in Acute Stroke Treatment; TACI: total anterior circulation infarcts; PACI: partial anterior circulation infarcts; POCI: posterior circulation infarcts; LACI: lacunar infarcts; MII-1: multi-inflammatory index 1; MII-2: multi-inflammatory index 2; MII-3; multi-inflammatory index 3

	Simple logistic regression		Model 1		Model 2		Model 3	
	OR (95% CI)	P-value	OR (95% CI)	P-value	OR (95% CI)	P-value	OR (95% CI)	P-value
Age, years	1 (0.98-1.02)	0.846						
Gender, male (female: Ref.)	0.49 (0.3-0.81)	0.005						
Smoking, smoker (non-smoker: Ref.)	1.06 (0.54-2.09)	0.871						
Hypertension, present (absent: Ref.)	1.19 (0.73-1.95)	0.482						
Diabetes, present (absent: Ref.)	2.44 (1.48-4.01)	<0.001	2.57 (1.35-4.88)	0.004	2.45 (1.29-4.66)	0.006	2.51 (1.32-4.79)	0.005
Hyperlipidemia, present (absent: Ref.)	0.52 (0.18-1.49)	0.222						
Coronary artery disease, present (absent: Ref.)	0.83 (0.46-1.48)	0.521						
MI/CVE, present (absent: Ref.)	0.59 (0.31-1.12)	0.109						
AF, present (absent: Ref.)	1.01 (0.59-1.74)	0.966						
Chronic renal failure, present (absent: Ref.)	6.05 (1.8-20.35)	0.004						
GCS score								
≤8	36.04 (16.73-77.63)	<0.001	23.13 (9.65-55.46)	<0.001	20.44 (8.65-48.31)	<0.001	21.2 (8.85-50.78)	<0.001
9-12	3.3 (1.53-7.1)	0.002	2.25 (0.98-5.19)	0.056	1.85 (0.8-4.25)	0.147	2.1 (0.92-4.8)	0.078
13-15 (Ref.)	-	-						
NIHSS score								
1-4 (Ref.)	-	-						
5-14	5 (1.13-22.02)	0.033						
15-20	48.54 (11.26-209.2)	<0.001						
≥21	122.85 (25.11-601)	<0.001						
TOAST								
Large artery atherosclerosis (Ref.)	-	-						
Cardioembolism	0.94 (0.53-1.66)	0.828						
Small vascular obstruction	0.26 (0.06-1.12)	0.071						
Other etiology	0.51 (0.19-1.38)	0.187						
No known cause	0.78 (0.34-1.79)	0.559						
Bamford								
TACI	7.5 (3.48-16.2)	<0.001	3.16 (1.23-8.1)	0.017	3.42 (1.33-8.83)	0.011	3.44 (1.32-8.97)	0.011
PACI	1.08 (0.5-2.34)	0.842	1.47 (0.59-3.64)	0.406	1.49 (0.6-3.7)	0.389	1.52 (0.61-3.8)	0.373
POCI (Ref.)	-	-						
LACI	0 (0-0)	0.998	0 (0-0)	0.998	0 (0-0)	0.998	0 (0-0)	0.998
MII-1	1.01 (1.01-1.02)	<0.001	1.01 (1.01-1.02)	0.001				
≥ 32.095 (<32.095: Ref.)	3.51 (2.1-5.87)	<0.001						
MII-2	1.01 (1-1.01)	<0.001			1.01 (1-1.01)	<0.001		
≥ 888.95 (<888.95: Ref.)	3.05 (1.81-5.14)	<0.001						
MII-3	1.01 (1-1.01)	<0.001					1.01 (1-1.01)	<0.001
≥17528.39 (<17528.39: Ref.)	4.01 (2.42-6.64)	<0.001						
Model performances								
AUC (95% CI)			0.885 (0.844-0.925)	<0.001	0.884 (0.843-0.925)	<0.001	0.887 (0.846-0.927)	<0.001
Cut-off point; SE, SP			0.233; 73.42, 89.81		0.153; 78.48, 86.33		0.273; 74.68, 90.62	

Parameters were separated according to the cut-off point determined by ROC analysis and analyzed in two types as continuous and categorical variables in logistic regression analyses. The simple logistic regression and multiple linear regression analysis results related to mortality are shown in Table 4. The performances of multiple models developed for the mortality cut-off points were evaluated. Together with other factors, Model 1 included the MII-1, Model 2 the MII-2, and Model 3 the MII-3. Although there was no significant difference between the AUC values of the models (DeLong test: p>0.05 for all comparisons), the AUC values obtained from largest to smallest were 0.887 (0.846-0.927) for Model 3, 0.885 (0.844-0.925) for Model 1, and 0.884 (0.843-0.925) for Model 2. The highest sensitivity rate was reached with Model 2 (74.48%), and the highest specificity rate with Model 3 (90.62%). The mortality risk was determined to be increased 1.01-fold (95% CI: 1.01-1.02, p=0.001) with a 10-unit increase in MII-1, by 1.01-fold (95% CI: 1.0-1.01, p<0.001) with a 100-unit increase in MII-2, and by 1.01-fold (95% CI: 1.0-1.01, p<0.001) with a 1000-unit increase in MII-3.

The factors with a significant effect on mortality were determined to be diabetes comorbidity, GCS groups, and Bamford classification. The mortality risk in Models 1, 2, and 3 was determined to be increased 2.57-fold (95% CI: 1.35-4.88, p=0.004), 2.45-fold (95% CI: 1.29-4.66, p=0.006), and 2.51-fold (95% CI: 1.32-4.79, p=0.005), respectively for patients with diabetes comorbidity; 23.13-fold (95% CI: 9.65-55.46, p<0.001), 20.44-fold (95% CI: 8.65-48.31, p<0.001), and 21.2-fold (95% CI: 8.85-50.78, p<0.001), respectively for patients with GCS ≤8 compared to patients with GCS of 13-15; and 3.16-fold (95% CI: 1.23-8.1, p=0.017), 3.42-fold (95% CI: 1.33-8.83, p=0.011), and 3.44-fold (95% CI: 1.32-8.97, p=0.011) respectively for patients in the TACI group compared to patients in the POCI group.

## Discussion

The results of this study demonstrated for the first time that the MII can be an independent predictor of mortality in ischemic stroke cases. In addition to this new finding, the study found that high WBC and platelet counts, as well as GCS and mRS, were all independent predictors of mortality, which aligns with previous research. The most important finding of the study, which will be valuable for clinicians in practice, is that MII can be a potent and independent index in determining stroke severity when compared to the previously used blood-related indices such as CRP and neutrophil count.

Hemogram-based inflammatory parameters have been previously investigated in stroke patients. For example, a previous study found that high WBC and platelet values were associated with mortality [17]. The results of the current study have consistently shown that these hemogram-based parameters are much higher in cases of mortality than in survivors. CRP is a positive acute-phase protein that has been associated with stroke prognosis. High CRP levels, as in many other disorders, are associated with poor prognosis and mortality in stroke patients [7,11]. As per the findings of the present study, the CRP levels were also significantly higher in non-surviving stroke cases compared to survivors. Various prognostic ratios such as NLR, PLR, and CLR provide insights into the prognosis of critical illness as well as ischemic stroke cases [11,17-19]. In this study, NLR, PLR, and CLR were significantly different in the non-surviving cases.

This study was mainly designed to examine the predictive potential of MII, a new hematological indicator, as a mortality indicator in ischemic stroke. MII was first described by Casadei Gardini et al. as a newly created prognostic index [8]. It was found to be helpful in determining the prognosis among colorectal cancer patients in their study [8]. In another recent study, Boyuk suggested that this new index could be helpful in distinguishing between massive and non-massive pulmonary embolisms [9]. Another study performed by Gozdas et al. on the association between MII and ICU mortality in COVID-19 patients reported that it may be an independent predictor of in-hospital mortality [7]. The current study showed that patients in the poor-outcome (mortality) group had significantly higher multi-inflammatory indices compared to those in the survivor group. It was also observed that increased stroke severity was associated with a higher MII. The PLT, NEU, LYM, PLR, NLR, and MII 1-2-3 levels in the group with poor outcomes were significantly higher than in the survivor group. Moreover, in the multivariate regression analysis, MII-3 was shown to be the most important of the multi-inflammatory indices and was selected as the most meaningful predictive hematological index for mortality. This result is different from ta of an MII-2 study involving a COVID-19 patient group in the literature [7]. The most interesting and significant finding of the current study is that for the first time in the literature, MII was found to be an independent predictor of poor outcomes after stroke. MII-3 had a higher rate of both specificity and sensitivity than the others. We believe that this feature makes MII-3 superior to other predictors. Therefore, MII-3 may be a potential predictor for stroke severity and mortality from AIS. Determining risk factors that predict poor outcomes after stroke is critical as it enables the creation of preventive strategies and implementation of rapid management when necessary. Many risk factors that can be used to predict poor outcomes have been found, and several risk indexing and modeling studies have also been conducted.

In this study, it was observed that the MII 1-2-3 value increased together with decreasing GCS and increasing NIHSS scores. Similar findings can be seen in the studies by Kara et al. [20] and Yao et al. [21], in which it was reported that stroke severity was significantly higher in AIS patients with a negative outcome compared to those with positive outcomes. It was seen that while both NLR and PLR may be promising indicators for predicting the functional prognosis of AIS, they may also help to identify patients more strongly at risk with higher values associated with MII and a higher risk of poor functional outcome in ischemic stroke patients. Regarding mortality, MII-3 was better than the other two indices when evaluated alone. In addition, as seen in Table 4, the success rate was higher in MII-3 when a mortality predictive model was created using other factors and MII. Although the best modeling was obtained with MII-3, there was no significant difference between indices. In order to make a distinction between the indices, studies with larger samples need to be conducted.

This study has a few limitations. Primarily, it had a homogeneous patient population as it was a single-center study. There was no control group for comparison. Including an age- and sex-matched control group could help correlate index values in stroke patients. In addition, the study, which included the duration of patients' admission to the hospital, could have made these findings more valuable. The inclusion criteria were restricted, and many factors can determine the prognosis of stroke. Also, since it was a retrospective study, the data could not be shown in detail.

## Conclusions

Based on our findings, MII is a simple and practical biomarker that can help in the early detection of poor prognosis in AIS. NLR was readily derived from PLR and CRP and was superior to neutrophils, lymphocytes, or CRP alone in distinguishing fatal cases of AIS. As MII is a new index, more supporting evidence is needed to guide its clinical application, and more comprehensive and large-scale studies are needed to confirm these results. Therefore, physicians should be more careful in the management of AIS cases with increased MII levels.
